# Diagnostic error will bring about a new era of digitalized medicine

**DOI:** 10.1002/jgf2.233

**Published:** 2019-01-19

**Authors:** Koichiro Nakano, Takashi Watari, Kenichiro Akazawa, Izumi Kitagawa, Satoshi Watanuki

**Affiliations:** ^1^ Division of Emergency Medicine Shonan Fujisawa Tokushukai Hospital Kanagawa Japan; ^2^ Postgraduate Clinical Training Center Shimane University Hospital Shimane Japan; ^3^ Division of General Internal Medicine Shonan Fujisawa Tokushukai Hospital Kanagawa Japan; ^4^ Division of General Internal Medical Department of Internal Medical Shonan Kamakura General Hospital Kanagawa Japan; ^5^ Division of Emergency and General Medicine Tokyo Metropolitan Tama Medical Center Tokyo Japan

To the editor,

We attended the 11th Annual International Conference of Diagnostic Error in Medicine (DEM) through 4‐6 November 2018 in New Orleans, LA. In the DEM conference, we discuss how to improve practice, because diagnostic errors cause many deaths.^1^ The DEM conference is held annually around the United States; this year's theme is “Innovating to Improve Diagnosis.”

Dr. Robert Watcher discussed how technology has changed medicine. Introduction of electrical medical records (EMR) has made access to test results and past records easier.^2^ However, we have only replaced our analog jobs with digital ones; the doctor looks at the screen more than the patient, increasing our burden and reducing patient satisfaction. Henceforth, we must create a new style of work using digitalized medicine.^3^ Analyzing data has become easier after digitalization. For example, using EMR, a physician can check glucose levels of an entire hospital in an hour. Artificial intelligence (AI) diagnosis is being tested in radiology and dermatology, as images comprise a mega databank for referral. Teaming up with major technology companies may lead us to great new innovations. However, we must keep in mind that the best fuel to energize this evolution process is our desire for better and safer diagnosis.

New technology is not the only way to improve our practice. We must encourage patients to join the diagnosis process. As there is so much information on the Internet about medicine, patients tend to want exact answers, but that is not the way medicine actually works. We must be open to patients about the uncertainty of medicine and ensure their participation, as only they can provide certain details. Moreover, this year, the Society to Improve Diagnosis in Medicine (SIDM) has launched an initiative called ACT for Better Diagnosis^TM^. The SIDM states that accuracy, communication, and timeliness of diagnosis must be improved; to achieve this, we must see what is blocking the improvement. Examples of blocks are incomplete communication, lack of feedback, limited support, limited time, and limited funding. Improvement in these areas will surely improve our diagnosis.

The most important thing we have learned is that our motivation to improve our diagnosis is the key for innovations in medicine. We should work with patients to reduce diagnostic errors; combined with new technologies, this will surely produce a new style of medicine. One never knows how we might be working in 10 years.

## CONFLICTS OF INTEREST

The authors have stated explicitly that there are no conflicts of interest in connection with this article.
